# Qualitative perspectives of early surgeon users on the value of the daVinci 5 surgical system

**DOI:** 10.1007/s11701-026-03366-w

**Published:** 2026-05-21

**Authors:** Derek J. Erstad, Zahra A. Fazal, Karlis Draulis, Feibi Zheng, Gretchen Jackson, Christy Y. Chai

**Affiliations:** 1 Division of Surgical Oncology, Baylor College of Medicine, 2002 Holcombe Blvd, OCL 112, Houston, Texas 77030 USA; 2 General Surgery and Surgical Oncology, Michael E. DeBakey VA Medical Center, Houston, Texas USA; 3 Intuitive Surgical Inc, Sunnyvale, California USA; 4 Department of Pediatric Surgery, Vanderbilt University Medical Center, Nashville, Tennessee USA

**Keywords:** da Vinci 5, Qualitative research, Surgeon experience, Robotic surgery, Force feedback

## Abstract

To understand the value and experience of using the da Vinci 5 (dV-5) robotic surgical system among early-adopting surgeons in the United States. In March 2024, da Vinci 5 was released with new features such as a haptic technology (called Force Feedback) and Case Insights, a tool leveraging artificial intelligence (AI) to deliver video recordings of cases with objective metrics of performance. Few studies have assessed the value and challenges experienced by surgeons using this new system. Twenty-three semi-structured qualitative interviews were completed with surgeon-participants over video conferencing software representing a selection of surgical specialties, case volumes, and practice types. Interviews were recorded, transcribed verbatim, and deidentified. Results were analyzed by one reviewer using an inductive-deductive thematic approach and further verified by another reviewer. Themes were mapped to value domains and challenges with adoption. Among the participants, there were a higher proportion of males, surgeons who practiced at community hospitals, and those with medium to high volumes of robotic cases. Thematic analysis revealed two main themes with seven subthemes exploring either the value beliefs or barriers/challenges with adoption to the new system. Participants found the most value in dV-5 with its ergonomic comfort, ability to support future training of surgeons, and an economic benefit in reducing operative time. There were mixed findings around its impact for improving clinical outcomes given the early system maturity. Thematic analysis of interviews with early-adopting surgeons indicated that the dV5 system supports better ergonomics, surgeon training and may have implications for some key surgical metrics such as tissue tearing and operative time.

**Supplementary Information:**

The online version contains supplementary material available at 10.1007/s11701-026-03366-w.

## Introduction

Over the last decade, there has been rapid adoption of robotic-assisted surgery (RAS) as a minimally invasive surgery (MIS) option [1]. RAS is now commonly applied across various surgical specialties including general surgery, gynecology, and urology [2, 3]. The da Vinci Surgical System (dV) has been used in over 10 million surgeries worldwide and is now recognized as a standard treatment for certain procedures in the United States [4, 5]. Numerous studies have documented the clinical benefits associated with RAS, including reduced hospital length of stay, pain, and surgical site infection compared to open surgery [6–10]. More recently, RAS has also been associated with improved access for marginalized groups to MIS treatment options [11–13]. 

In March of 2024, the latest of Intuitive, Inc.’s systems called the da Vinci 5 (dV-5) was launched within the US market, introducing new features including force feedback, integration of artificial intelligence (AI), improved ergonomic comfort, and high-definition imaging [14]. These features have yet to be evaluated for their impact on clinical, economic and humanistic outcomes. However, early evidence from select procedures indicates that enhanced visualization and force feedback technology may be associated with modest improvements in clinical and perioperative outcomes [15–17]. Furthermore, the new platform is associated with improved ergonomic support for surgeons, which has previously been a challenge [18, 19]. These innovations may influence aspects of surgical practice, including ergonomics, surgical workflow, training and mentorship, and potential clinical outcomes.

To date, most published literature on the value of RAS and specifically the dV system has focused on quantitative and database studies. Few qualitative studies have assessed the experience of nurses, physicians and patients with dV systems [20–22]. Given the limited literature on user-centered perspectives, we sought to evaluate the perspective of early surgeon users to assess the claims of potential impact with the dV-5 system. Specifically, our research question was:

1. How do early-adopting surgeons perceive and define the value tenets of the da Vinci 5 system?
2. What are the barriers associated with adoption of the da Vinci 5 system and its associated Case Insights platform?

Understanding surgeon perspectives in early adoption may provide insight into how these features influence surgical practice and identify areas of value and barriers to adoption. This study leverages qualitative research methodologies to explore early adopting surgeons’ experiences with dV-5, assessing both its potential novel value, barriers to adoption, and new system challenges.

## Methodology

### Study design

A qualitative descriptive design was used, employing a grounded theory-informed thematic analysis. Rather than generating a formal theory, this framework was used to identify themes and organize them into a conceptual model [23]. The approach explored how the features of dV-5 potentially deliver clinical, economic, and humanistic benefits across diverse healthcare settings, focusing on the experiences of surgeons as early adopters. A qualitative design allowed for a contextual exploration of surgeon experience including both the value, barriers, and challenges that they have found with adopting the new robotic system [24]. This method was chosen over quantitative approaches due to the ability to uncover motivation in surgeon decision-making, and nuances in discussions of perception of value. To the best of our knowledge, this study is the first to explore the value of dV-5 among early adopting surgeons across different practice settings.

### Participant selection

The target population was surgeons in the United States from any specialty background that used the dV-5 system. The specific inclusion criteria were that surgeons had at least 10 dV-5 cases completed, and a lifetime of 50 or more robotic cases completed at the time of recruitment. These criteria allowed for a selection of surgeons with enough dV-5 experience who could compare this latest robotic system to previous robotic systems and other forms of minimally invasive surgeries. Additionally, this allowed for a large sample size of surgeons to recruit from given the conservative dV-5 case estimate.

A master list of eligible surgeons was created using an internal surgeon registry (*N* = 753) provided by Intuitive Surgical, to allow for identification of surgeons who met the inclusion criteria of having used both robotic platforms in the aforementioned quotas, and to further characterize participants by sociodemographic and surgeon-specific variables available in the database. Next, code was developed to randomly sample participants from this master list with an equal distribution of surgical specialty, case volume, and practice type. This was done to create a subset of participants to invite for recruitment while minimizing investigator’s sampling bias in its determination. This resulted in a sample of 100 participants who were then invited for recruitment. This quasi-random purposive sampling technique allowed for a balance between identifying surgeons who met our eligibility while ensuring a diverse distribution of specialty, experience (robotic and dV-5 case volume), and practice type (academic/community hospital) to the extent possible. Ultimately, invited participants who consented and were available for the interviews were enrolled in the study.

**Fig. 1:**
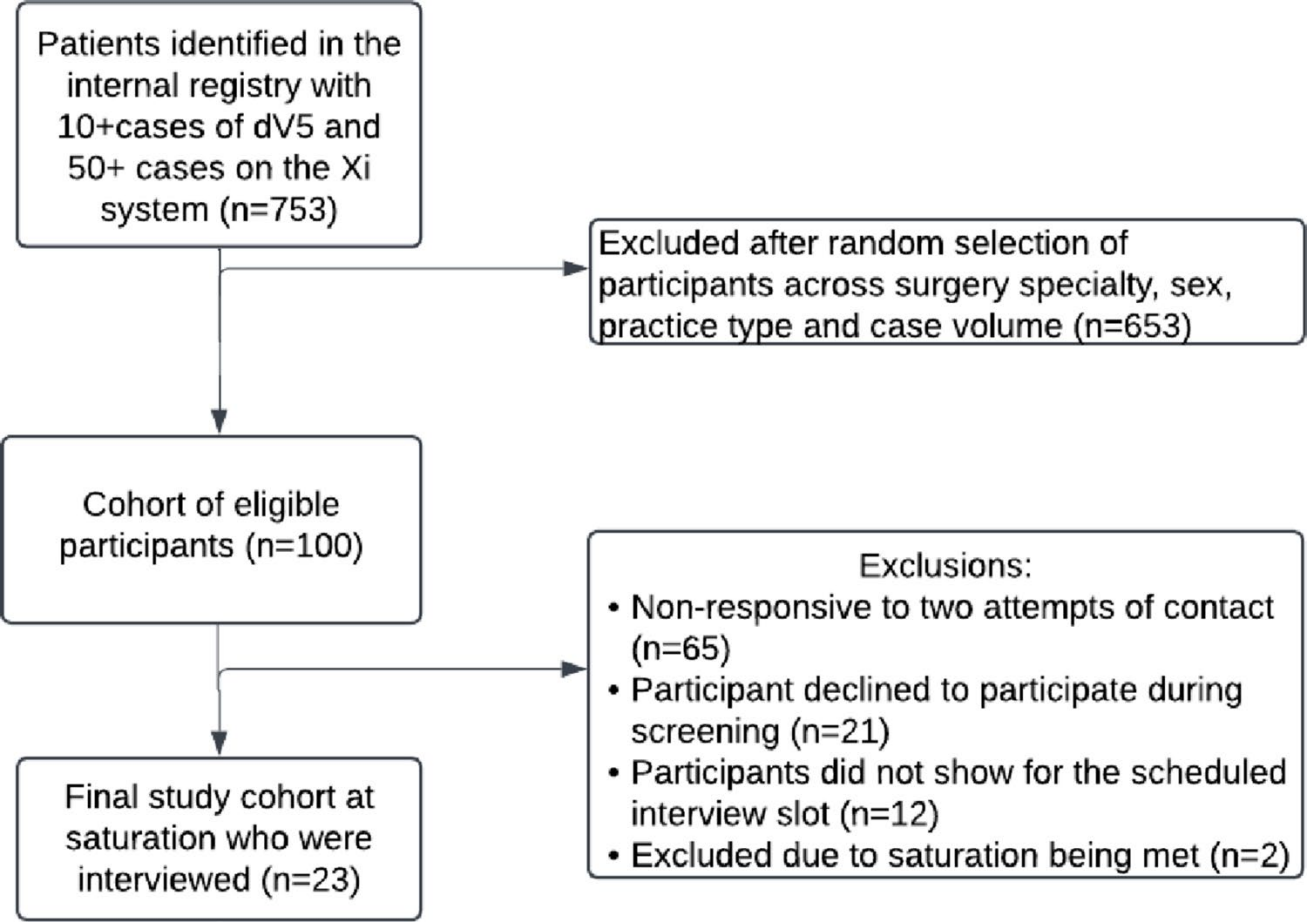
Flow diagram of cohort selection. Descriptive caption: A flow diagram that demonstrates the initial sample size of 753 participants who were identified in an internal registry to meet the inclusion criteria, and were then sampled down to 100 participants randomly selected across sex, specialty, practice type and case volume. The final study cohort included only 23 participants form the initial 100 with exclusions due to non-response, drop-out, and saturation being met

Following WCG Institutional Review Board (IRB) approval (20233310 on 11/2024), initial contact via email was made by a contracted market research vendor to minimize recruitment bias from having recruitment associated with a specific robotic device company. A week after the initial contact by the vendor, an email reminder was sent followed by a phone call a week later as needed. All participants were offered an honorarium of $200, paid by the vendor directly and funded by Intuitive Surgical, as the rate set to reflect fair market value for surgeon time. Interview scheduling continued amongst participants who consented until content saturation was achieved. Response rate from the contact efforts and dropout rate after eligibility was recorded and is represented in a flow chart (Fig. 1).

### Data collection

Participants who responded to the study invitation were scheduled for a 45-minute semi-structured interview over a video conferencing software (Zoom) with author ZAF (female), formally trained in human subjects’ research and who had prior experience with qualitative data collection. The non-clinical background of the interviewer was considered advantageous in minimizing hierarchical bias during data collection with surgeons. Interviews were chosen over focus group discussions due to their ability to be in-depth and reduce power dynamics in conversations, especially amongst low versus high volume surgeons. Additionally, the semi-structured interviews provided opportunities for the participants to speak to the value pillars through their own practical experience beyond the pre-set questions. An interview guide was used to collect background information about the participant and included pre-set interview questions that were designed collaboratively by members of the research team, and approved by a senior surgeon (Supplemental files 1). Questions in the interview guide were informed by the current gap in peer reviewed literature [15–17] and market research. Although the guide was not pilot tested with participants, it underwent structured internal review with team members who had expertise on the product, clinical practice and qualitative methods.

Before the start of an interview, participants underwent an informed consent process with author KD to understand the study’s purpose, procedures, and potential risks and benefits. Interviews were audio and video recorded and then transcribed verbatim. No other non-participants were present during the interviews, and no prior relationship was established with interviewees. All personal health information was de-identified from interviews after transcription to protect confidentiality. The ‘consolidated criteria for reporting qualitative research’ (COREQ) checklist was used to report this study [25]. 

### Data analysis

A hybrid inductive-deductive thematic approach was used to analyze the interview transcripts combining pre-existing hypothesized themes with emergent themes from participants [24]. Both transcription and analysis were conducted using MAXQDA software [version 24, VERBI, Germany]. A codebook was developed before interviews were conducted whereby an initial list of codes, descriptions and examples were generated to capture themes including clinical, humanistic, economic and surgeon-specific value. These deductive themes were informed by the study objectives and the conceptualization of RAS value within previous literature [6, 15]. After transcription of each interview, open coding was performed by one reviewer (author ZAF) to identify emergent ideas directly from the data. Each of these inductive themes were then categorized into parent deductive themes. Through team discussions and validation by a senior surgeon, themes were refined to ensure relevance and accuracy. Content saturation was monitored by the primary coder (author ZAF), and confirmed by team discussions, in parallel to thematic analysis. Saturation was operationalized as three consecutive interviews in which no new codes or variations in existing themes were revealed.

At the end of the first round of analysis, a subset of interviews were re-assessed for verfication of coding structure by another reviewer (author KD) to ensure credibility of analysis. Any coding discrepancies were then resolved through consensus. Finally, themes were compared against existing literature to contextualize findings and uphold triangulation. The steps for data analysis are summarized in the a priori determined protocol (Supplementary files 2).

### Reflexivity statement

The research team consisted of members with minimally invasive surgery expertise (CC, DE, FZ, GJ), qualitative research expertise (ZF, GJ and KD), health informatics training (GJ) and experience with evaluating health information technology (GJ and KD). The qualitative researchers led the data collection and analysis while surgeons provided insight in developing the interview guide, oversight during the study stages, and contextual interpretation of findings through their clinical experience. The senior author (CC) had final say on key study components including data interpretation and manuscript approval.

Assumptions about surgical technology may have shaped the data collection and analysis. Although the study involved Intuitive Surgical personnel, all members were from research rather than commercial/marketing teams. Regardless, considerations around bias were addressed wherever possible. Mitigation strategies employed included sampling across diverse surgeon characteristics (sex, practice type, case volumes, and specialty), verification of themes, literature feedback loops, and systematic reflexivity throughout the study. Additionally, the interview guide was developed prior to recruitment and chosen to be semi-structured to allow participants to guide the discussion. During the analysis, themes that emerged were documented in the codebook and iteratively classified through consensus discussions to ensure alignment with findings rather than researcher’s assumptions. Finally, we intentionally chose a hybrid inductive-deductive thematic analysis approach to reflect a balance between a systematic evidence analysis and openness to emergent themes, fostering transparency and co-ownership of the findings.

### Ethical considerations

The study was reviewed and approved as a sub-study amendment under the parent protocol by WCG IRB (approval number: 20233310). The study prioritized confidentiality and anonymity of participants by using encrypted data storage and anonymizing responses in the final analysis. Participants were fully informed about the purpose, methods, and potential implications of the study through a detailed informed consent process. They had the right to withdraw from the study at any point, and data was stored in compliance with relevant regulations, including the Health Insurance Portability and Accountability Act (HIPPA).

## Results

### Participant demographics

The final cohort consisted of 23 surgeon participants, with a higher proportion being male, practicing in community hospitals, and from the general surgery subspecialty. Case volume cut-offs were created using quartiles from the master list with dv-5 volume thresholds being 0–20,21–40, 41–60, and 61 or more while RAS thresholds being 0-500, 501–800 and 801 or more, respectively for low, medium and high volume. Participant characteristics are summarized in Table 1.

**Table 1:** Baseline characteristics of study participants

Characteristics	Sample size, *N* (%)
Sex	
Female	5 (21.7)
Male	18 (78.3)
Practice type	
Community	17 (73.9)
Academic	3 (13.0)
Other	3 (13.0)
Experience level (RAS only)	
Less than 10 years	16 (69.6)
More than 10 years	7 (30.4)
dV-5 volume	
Low ( < = 20)	5 (21.7)
Medium Low (21–40)	7 (30.4)
Medium High (41–60)	7 (30.4)
High ( > = 61)	4 (17.4)
RAS volume	
Low ( < = 500)	8 (34.8)
Medium (501–800)	6 (26.1)
High ( > = 801)	9 (39.1)
Surgeon specialty	
Obstetrics and Gynecology	5 (21.7)
Gynecological Surgery	1 (4.3)
General Surgery	15 (65.2)
Thoracic	2 (8.7)

_RAS – Robotic−assisted Surgery; dV−5 – da Vinci 5 surgical system_

### Overall findings

Thematic analysis revealed two overarching themes: 65.6% of coded segments were characterized as value beliefs while 34.4% were characterized as challenges or barriers with the technology. Within each of these themes, several subthemes captured the distinct experience of surgeon users with the technology. Table 2 presents the coding framework including the two overarching themes, subthemes and associated frequency across participant interviews. Consistent with qualitative principles, frequency and stratifications are reported only to orient the distribution of perspectives and explore emerging patterns but does not imply statistical representativeness or conceptual importance. Narrative interpretation was supplemented by in-depth quotes for each subtheme. When stratified by surgeon volume of participants, a heat map revealed association between subthemes (Supplemental files 3). Notably, low-volume surgeons reported an increase in operational efficiency due to dV-5 and a greater degree of ergonomic comfort. In contrast, medium and high-volume surgeons reported more challenges in operationalizing the value of force feedback technology and found it to be useful for training/mentoring but not directly applicable to their own practice. Finally, high-volume surgeons also reported an increase in autonomy and consequently a decrease in operative time due to the system consolidation of features.

**Table 2:** Frequency of themes and subthemes

	Inductive theme	Description of theme	Deductive subthemes	Frequency (%)
	1. Surgeon value	Perceived benefits experienced by surgeons rather than patients or hospitals	• Ergonomic comfort of the system • Application to training/mentorship of surgeons • Increase in surgeon autonomy • Self-improvement using data metrics	25.8
	2. Economic value	Perceived financial benefits or system-level value associated with dV-5 adoption	• Increase in operational efficiency • Increase in patient throughput • Sustainability and modularity of the system • Support in performing complex cases • Decrease in OR time	18.6
	3. Clinical value	Insights into the clinical advantages of the dV-5 system	• Decrease in LOS • Decrease in SSI/tissue tear • Decrease in blood loss • Less pain prescriptions • Safety/quality checks for clinical outcomes due to Case Insight data	13.5
	4. Humanistic value	Perceived impact on the user experience, including patient-focused factors.	• More data for patient education and research purposes • Increase in accessibility of MIS for both patients and surgeons who are new to RAS • Faster return to life for patients	7.7
Challenges/barriers in dV-5 adoption	5. Provider level	Barriers stemming from individual surgeons’ or care teams’ attitudes, experiences, knowledge, or workflow concerns	• Interpretability of Case Insight data on objective performance indicators • Perceived relevance of force feedback technology • Limited translation of metrics into surgical decision-making and evidence for improved clinical outcomes	14.8
	6. Data infrastructure level	Barriers related to the technological systems and their analytic capacity	• System lag and/or instrument exchange delays • Gaps in data metrics recorded and device functionality	10.9
	7. System level	Barriers embedded in broader organizational, regulatory, economic, or policy contexts that influence adoption at a structural level	• Approval/roll-out lag in new technology • Learning curve for hospital staff/ administrators • Regulatory uncertainty around data governance and legal exposure	8.7

_dV−5 – da Vinci 5; LOS – Length of stay; MIS – Minimally Invasive Surgery; RAS – Robotic Assisted Surgery; SSI – Surgical Site Infection; OR – Operative Room_

### Subtheme 1: Surgeon value

There was consensus amongst all interviews for improvement in ergonomic comfort with the system’s new head-in feature and its contribution towards less neck tension, fatigue and perceived long-term well-being.

“Most of my cases are on the dV-5 and when I had to switch back to the Xi, I was able to tell a difference from an ergonomic standpoint. I’m young in my career, and want to operate for another 30 years, so noticing the difference in my neck positioning and the kind of flexion that I’m having to incorporate are important to me” – (S18, Male, General Surgery, high dV-5 volume, medium lifetime RAS volume).

“I like the console adjustments, and the ergonomics [of the dV-5 system] are just better. I noticed that I do not have neck strain and problems after a day of operating. If I am doing a longer case, I will notice things like that - (S12, Female, General Gynecology, low dV-5 volume, low lifetime RAS volume).

Participants also emphasized the increase in their autonomy and control within the operating room with the consolidation of features in dV-5, particularly in settings with less experienced support staff.

“I’ve always dropped my pressure after I got my ports placed, and so not having to ask someone else to do it or having to walk across the room to drop the pressure, and instead doing that while seated has been great” – (S10, Female, General Gynecology, medium high dV-5 volume, medium lifetime RAS volume).

“We have some great nurses, and some less familiar nurses. When I have someone great, I may not even get the chance to do things like adjust pressure, because they’ve already done it but some of the others it’s quite helpful to be able to be your own help instead of needing to rely on more people” – (S10, Female, General Gynecology, medium high dV-5 volume, medium lifetime RAS volume).

Additionally, participants noted the potential for various features of the system in improving training opportunities for new robotic surgeons, from better visualization in dV-5 to the use of metrics for assessment during surgical education certification in residents.

“Having the video recordings immediately available is valuable in case perioperative complications occur. I can look back at a certain part of the case [for self-improvement] or I ask my more senior partner by showing them the video.” – (S12, Female, General Gynecology, low dV-5 volume, low lifetime RAS volume).

“I am a reviewer for C-SATS (a video-based surgical assessment system), so I see the benefit of reviewing video content and getting an expert opinion on qualitative and quantitative metrics on that. I see that being integrated into this new platform” – (S7, Female, General Gynecology, medium high dV-5 volume, high lifetime RAS volume).

“I once did a robotic Whipple with a trainee, and I let him do the gastrojejunostomy. Afterwards he came to me and felt that his anastomosis was a little bit awkward. I tried drawing it on a whiteboard, but he had no idea what I was talking about because it’s a 2D representation. So, I brought up the video [on Case Insights] to point to what I thought he should have done vs. what he did” – (S23, Male, General surgery: Hepato-Pancreato-Biliary Surgery, low dV-5 volume, low lifetime RAS volume).

**Fig. 2:**
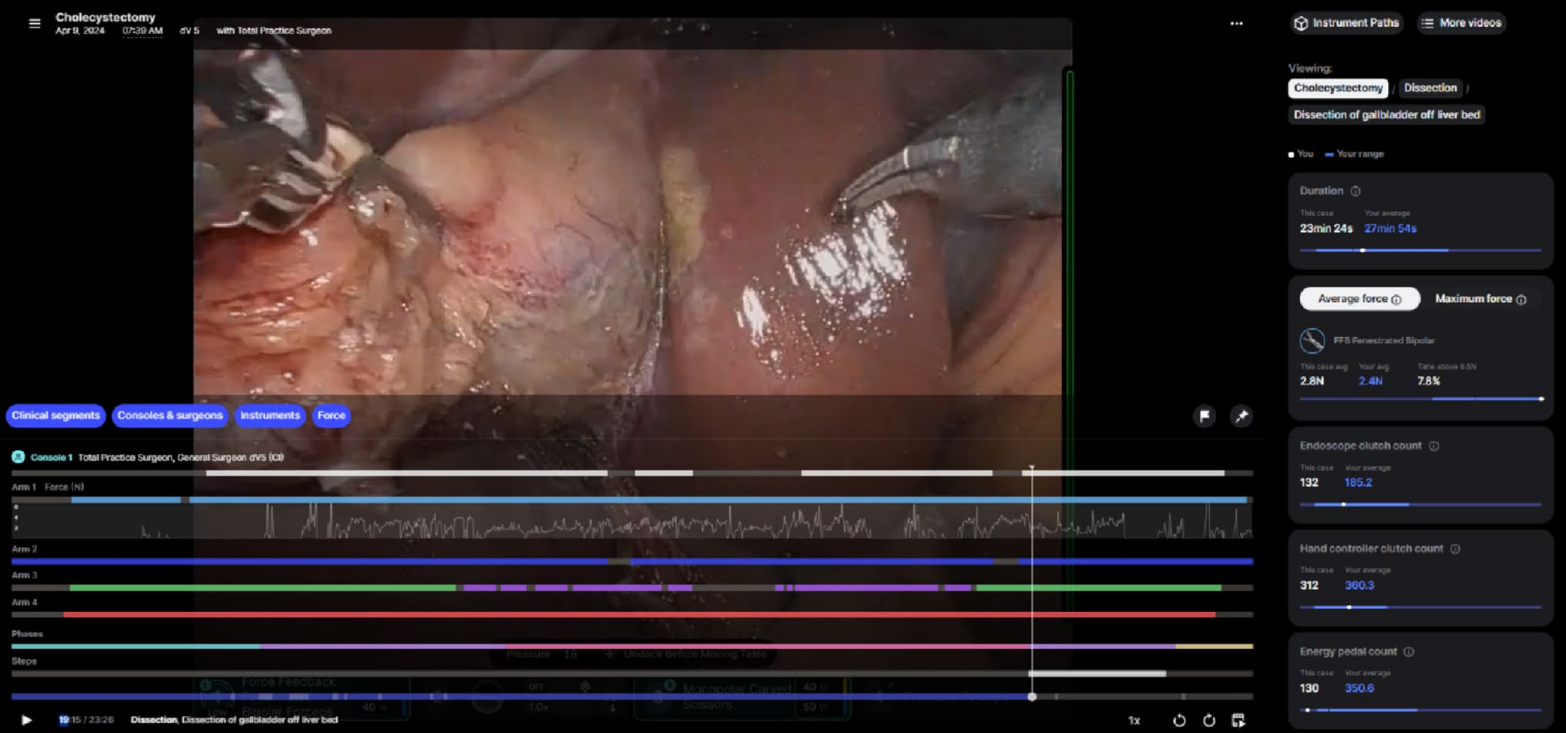
Case Insights platform. Descriptive caption: Screenshot of the Case Insights surgical analytics application displaying a video review snapshot of a robotic cholecystectomy during the transection phase. The main portion of the screen shows an intraoperative view of the gallbladder being dissected using robotic instruments. A timeline overlay beneath the video indicates activity from Console 1 and Arms 1 through 4, with color-coded bars showing instrument usage and activation over time. Additional segmented tracks display the procedure phases and individual steps. On the right-hand panel, case-level Objective Performance Indicators (OPIs) are listed, including total duration, average force with an instrument, endoscope clutch count, hand controller clutch count, and energy pedal count

Finally, the new data metrics included within Case Insights platform (Fig. 2) that comes with the dV-5 system were seen to be supportive of continuous learning due to its benchmarking ability and provided new opportunities for self-improvement including force applied during surgery.

“It has been helpful to look at the coordination of instruments for all the gynecologists who use robotics, and then standardized our pans to just one. We’re also able to see if someone’s below or above a certain standard deviation compared to the national average for operation times. That’s when we know to do case observations or provide more module training [to support their improvement]” – (S10, Female, General Gynecology, medium dV-5 high volume, medium lifetime RAS volume).

“The biggest contribution for the dV-5 over laparoscopy is that it flattened the learning curve for minimally invasive surgery. The movements are not as exaggerated as laparoscopy, they’re finer movements that used to take time to get used to and to train to feel how much you are pulling by seeing how everything around it extends. Now you’re removing layer by layer, the barriers to picking it (minimally invasive surgery) up” – (S23, Male, General Surgery: Hepato-Pancreato-Biliary Surgery, low dV-5 volume, low lifetime RAS volume).

### Subtheme 2: Economic value

**Fig. 3:**
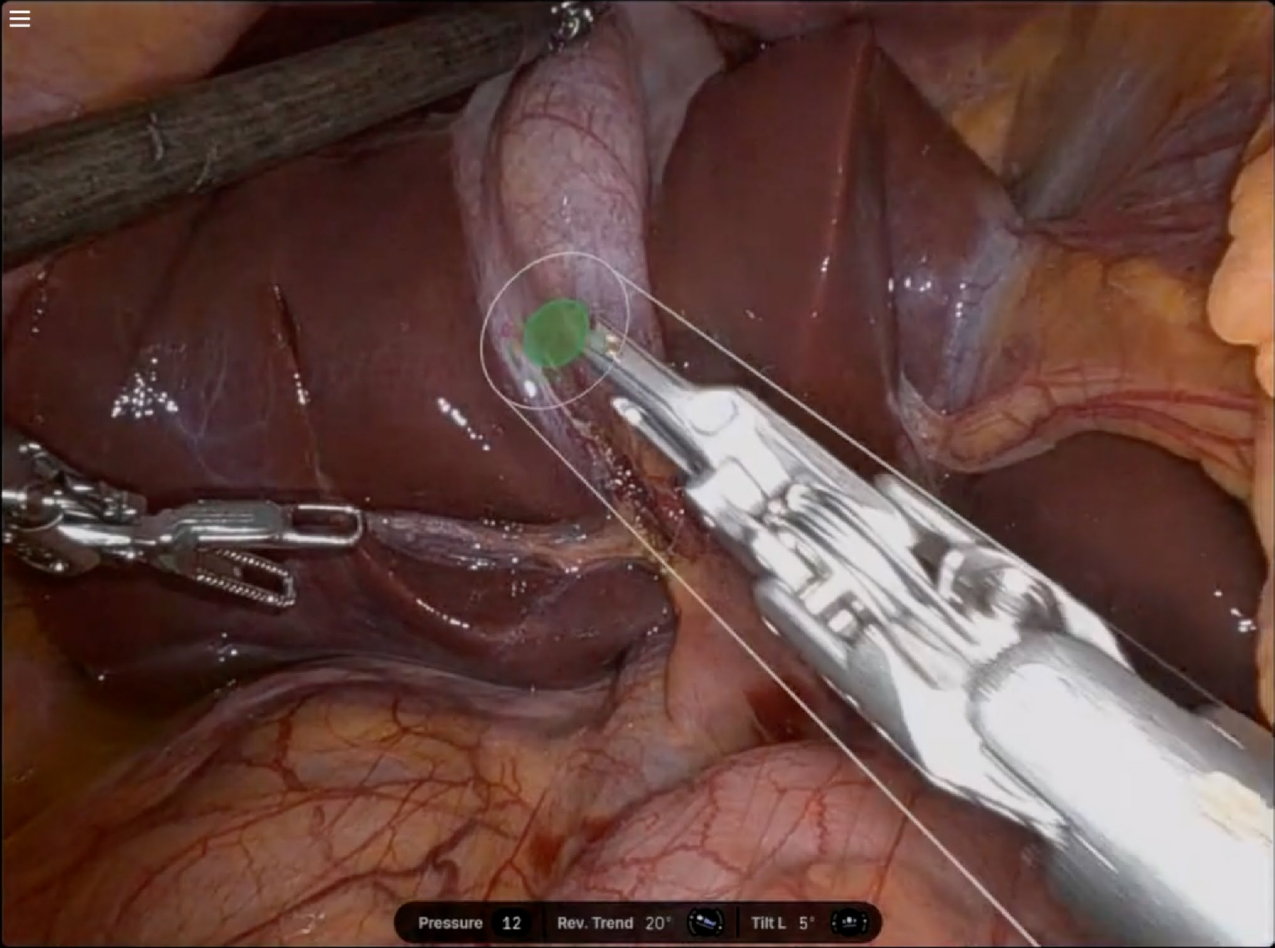
Guided instrument exchange. Descriptive caption: Screenshot of a robotic surgical console display during a guided instrument exchange in a cholecystectomy. The image shows an internal view of the liver and gallbladder region. A robotic instrument is centered in the frame, highlighted with a translucent circular target and alignment guides indicating the system’s guided exchange feature. On-screen visual overlays assist with positioning and alignment during the instrument swap. At the bottom of the screen, interface indicators display real-time metrics including pressure (12), reverse Trendelenburg angle (20 degrees), and tilt (5 degrees), providing situational awareness to the surgeon or operating room support staff during the exchange process.

The dV-5 system was associated with a perceived improvement of operational efficiency due to the features and instrumentation that were seen to support a streamlined workflow (Fig. 3) as well as better use of physical space in the operating room. Both these advantages contributed to anecdotal reports of a higher case turnover and lower operative time.

“The instrumentation cone makes things faster, because people know where the instruments should go and are not worrying about skewering something. It’s made my cases faster and saved me10-15 minutes for each case. That’s another hour for which I can do another surgery” – (S17, Male, General Surgery: Bariatrics, high dV-5 volume, medium lifetime RAS volume).

“The biggest expense of any hospital system is going to be the operating room, and from my personal experience, I went from having adrenals that stayed a couple of days to now stay overnight, and I went from cases laparoscopically that took me about 4 hours console time to 50 to 60 minutes for these procedures on the dV-5. That a cost saving metric” – (S6, Female, General Surgery: Endocrine Surgery, medium high dV-5 volume, medium lifetime RAS volume).

“Starting from the physical system, it’s self-contained and takes up less of a footprint in our operating room, which, you know, space is always a premium” – (S19, Male, General Surgery, low dV-5 volume, high lifetime RAS volume).

Some participants also noted that the dV-5 system enabled more complex surgery that would have been converted into open due to various features including the enhanced visualizations and force feedback technology providing haptics expanding case capabilities.

“I wasn’t doing big hiatal hernias with the robot because I was worried about tissue damage. Now, with the dv5, I’ve been tackling more of those. Specifically with the force feedback instruments, it lets you tackle more difficult cases you usually wouldn’t because you were worried about tissue injury but now, you’re gentler on the tissue, and you have better visualization of the anatomy” – (S2, Male, General Surgery: Bariatrics, medium high dV-5 volume, medium lifetime RAS volume).

### Subtheme 3: Clinical value

Given the limited quantitative data on the clinical outcomes associated with robotic surgery on the dV-5 system compared to other technology, participants hypothesized or referred to anecdotal evidence of improved outcomes or the ability to support intraoperative safety as a contributing factor for quality improvement.

“Being able to have 3D vision allows me to find the ureters and know exactly where they are at all times and dissect out little spaces. Whereas laparoscopically, you can’t see tiny nerves very well, or things that could be really consequential if you were to hit them” – (S12, Female, General Gynecology, low dV-5 volume, low lifetime RAS volume).

“There’s a fixed number of molecules of ATP that are going to be required in an operation. As a surgeon, it’s our job to cost the patient the fewest molecules of ATP by being more gentle and precise… I’m convinced from my own practice relative to others, that my care and delicate nature (with the support of force feedback), results in superior outcomes, fewer complications, and faster recovery. Everything that I have at my disposal to make me better at that is going to be better for the patient directly” – (S9, Male, Gereral Surgery, high dV-5 volume, high lifetime RAS volume).

“If you have less tissue trauma with force feedback then you’re going to have less edema, which is a big deal in bariatric surgery, because after bypasses, they [patients] can’t drink or eat, and that keeps them another day in the hospital. I also think controlling the pressure [the way the dV-5 system allows for] and being able to do the surgery under less pressure sometimes will lead to less pain because you’re inflaming the abdominal wall less” – (S17, Male, General Surgery: Bariatrics, high dV-5 volume, medium lifetime RAS volume).

### Subtheme 4: Humanistic value

The dV-5 system features were seen to facilitate humanistic outcomes such as increased accessibility of both patients and surgeon trainees to access minimally invasive surgery through telepresence while case videos and associated metrics were found to be useful in supporting patient education and shared decision making. Although these features are not limited exclusively to dV-5, the participants drew connections between them and their ability to impact value beyond the economic, clinical or surgeon-specific domains.

“I’ve shown [videos] to some patients who may have had a finding that have us make the interoperative decision that we shouldn’t go forward to explain them why we didn’t proceed” - (S3, Male, Gerneral Surgery: Bariatrics, high dV-5 volume, high lifetime RAS volume).

“As an endocrine surgeon being able to show people through the video recording, what in broad stroke adrenalectomies looks like has been helpful from a patient education experience” – (S6, Female, General Surgery: Endocrine Surgery, medium high dV-5 volume, medium lifetime RAS volume).

“Rural communities are definitely underserved, they just don’t have enough doctors. People can’t fly to some of these places so having an expert come and tele-proctor you on something, or watch you do a surgery would be a big win… The more people you train, it will give the people access to better care” – (S2, Male, General Surgery: Bariatrics, medium high dV-5 volume, medium lifetime RAS volume).

“We’ve got a handful of surgeons that if they’re going to convert, they’re going to convert, no matter what. But if we could get it [telepresence] to be used, where we try to ask for help before conversion on cases where there’s lots of scar tissue, then we would be able to give more patients better outcomes” – (S10, Female, General Gynecology medium high dV-5 volume, medium lifetime RAS volume).

### Subtheme 5: Provider-level barriers

Provider-level barriers were identified in the interpretation of metrics for new features including the Force Feedback and Case Insight technologies as well as concerns around not having enough clinically relevant data to allow for implementation of the findings from the data into everyday practice.

“I think the challenge is really learning how the data [from Case Insights] can help you change your practice” – (S17, Male, General Surgery: Bariatrics, high dV-5 volume, medium lifetime RAS volume).

“If you can correlate the degree of force with complications like vascular injury, tissue scarring, or other outcomes then you’re setting some sort of standard. [Example] You shouldn’t use more than X number of Newtons of force on this particular tissue when you’re doing lymph nodes or a hysterectomy, or when you’re dealing with the bowel. That would be helpful in training. We can’t feel the force of the robot, but we know that it’s strong and has no limit, so you can tear things very easily but we don’t know the threshold [of safe force] we can apply yet.” – (S8, Female, Gynecology: Gynecological Oncology, medium low dV-5 volume, low lifetime RAS volume).

### Subtheme 6: Data infrastructure barriers

The data infrastructure set of barriers included delays in computing or instrumentation exchange as well as unmet technological needs around device functionality. Additionally, interviews revealed insights into metrics yet to be captured with the current update of the system that would have been valuable for surgeons.

“There are significant delays occasionally when swapping instruments. You can tell that [the console] just stopped thinking/computing. Another example is that the smoke evacuator works well sometimes and other times not.” – (S22, Male, General Surgery, high dV-5 volume, high lifetime RAS volume).

“I think AI (artificial intelligence) can help with to determine [what parts of the video in case insights to cut and edit together]. I’m sure there’s products out there already that do it [stitching together a video]. But having it integrated intoCcase Insights would be pretty neat” (S13, Male, Thoracic Surgery, high dV-5 volume, high lifetime RAS volume).

“It’d be nice if there was some way to get AI generated feedback for specific points of the case and say, “Well, you get an A + for this portion of the procedure but on this portion of the procedure you only get a B minus, and these are all the things that you can do to improve. Having more actionable items is valuable” – (S7, Female, General Gynecology, medium high dV-5 volume, high lifetime RAS volume).

### Subtheme 7: System level barriers

Challenges at the hospital system level were identified among surgeons. Barriers of note included a learning curve for staff in the operating room (OR) in adjusting to the new system, and legal and regulatory challenges around data ownership for Case Insight videos.

“I don’t know where the archive of that video [from my Case Insights] is going to stay because it’s going to stay somewhere. I don’t think it’s going to be deleted completely [even when I delete it on my account]. I’m telling you this because it’s important, you might be giving more tools for the attorneys and for an expert witness. Believe me, even if the case looks perfect, you can find a defect; that’s for sure” – (S14, Male, General Gynecology, medium low dV-5 volume, high lifetime RAS volume).

”What many people are concerned about is what are the legal ramifications of, say, a surgeon who I’m not a partner with who calls me from some place in our hospital system and then I am documented in the note as someone who okay-ed the surgeon to do this procedure (or gave advice on it). Then, say there’s a complication. Even though I was not in the room, what is the legal responsibility of giving advice when someone is calling you and asking you for help [especially if something goes wrong.]” – (S8, Female, Gynecology Gynecological Oncology, medium low dV-5 volume, low lifetime RAS volume).

“I don’t really have to relearn anything major since the technology is very much the same. I sometimes have to remember the finger clutching to switch instrumentation since I use the Xi a lot where I am using the foot pedal to switch my instrumentation. It takes a little bit of mental preparation to remember that.” – (S17, Male, General Surgery: Bariatrics, high dV-5 volume, medium RAS lifetime volume).

## Discussion

In this qualitative study, the underlying value and barriers with adoption of the dV-5 system were explored among surgeons of different specialties, case volume and practices. From the twenty-three interviews, the most recurrent value-belief of dV-5 was cited at a surgeon-level (25.8%) which included improved ergonomics, increased autonomy due to system consolidation, and the use of data metrics for training and self-improvement. This was followed by economic (18.6%), clinical (13.5%) and humanistic (7.7%) value-beliefs. The most frequently cited challenges were identified at the provider-level (14.8%) followed by the data infrastructure (10.9%) and system levels (8.7%). Finally, when these subthemes were stratified by surgeon volume, low-volume surgeons ascribed more value to novel features of the dV-5, including force feedback technology, when compared to high-volume surgeons. This subjective observation may indicate that novel features of the dV-5 system provide value specifically for surgeons earlier on their learning curve for RAS.

Additionally, dV-5’s ergonomics was mentioned by both male and female surgeons to reduce strain, fatigue and support better body positioning. This could have particular significance in narrowing the gender gap in surgical ergonomics. Prior literature has documented disparities in ergonomic strain between male and female surgeons during non-robotic operations [26, 27]. This gender disparity exists even after controlling for confounding variables such as surgeon height and duration of operation, and has been associated with an increase in work-related injuries [27, 28]. Robotic surgery has been documented to decrease some of this strain including on the neck, back, hip, knee, ankle, foot and shoulder but previous version of robotic surgical systems were still associated with a gender difference in pain [29]. As more women enter the surgical workforce, ongoing enhancements in ergonomics—particularly those incorporated into the dV-5—are likely to have a positive impact on female surgeons, including during pregnancy, when ergonomic optimization is especially critical [30]. 

The predominance of training-related themes identified in this analysis is aligned with and underscored by previous literature. Gall et al., conducted a randomized control trial that found that surgical trainees performing robotic surgery had fewer suture errors and better physical comfort levels compared to trainees using laparoscopic surgery [31]. This is validated by surgeon quotes from our study that demonstrated the unique dV-5 features including video capture, objective performance metrics, force data and improved ergonomics had use-cases for self-improvement, teaching new residents and quality benchmarking. A systematic literature review found that such novel training methodologies incorporated in robotic system upgrades have been linked to positive effects on surgical proficiency [32]. 

Finally, the findings also pointed out the emerging value of Case Insights data which was used for patient education, self-improvement and training. In the past decade, the demand for clinically relevant performance metrics as part of robotic surgical training that can support procedure-specific learning has increased [33]. Studies have validated the use of such metrics against trained research staff and found a high degree of correlation suggesting that these measures are reliable for use in assessing surgeon skill and proficiency [34]. The dV-5 system further expands on this with force and instrument exchange data, thereby providing an enhanced basis for assessing surgeon proficiency. However, notable barriers in the use of Case Insights included limited understanding of how to interpret data metrics and their clinical relevance, as well as potential legal ramifications associated with storing surgical video data. This underscores the importance of data governance in promoting the wider adoption of digital products associated with surgical platforms.

Beyond the surgeon, the technological refinements of the dV-5 also empower non-surgeon team members. As one interviewee noted, “Usually if I wanted insufflation adjusted, I would need to call out, wait for them to hear my instructions and perform the action. This pauses the operation, disrupting the surgical flow, so being able to control pressure or smoke evacuation directly from the console reduces miscommunication, letting my team focus on their roles. Also, as the surgeon I can see and verify where they’re putting instruments in real time without having to pause and stick my head out” Since instrument exchanges are typically performed by trainees or other non-surgeon team members, these design improvements—with built-in safety checks—enhance team confidence, streamline workflow, and reduce cognitive load at the bedside. This is particularly valuable in rural or community hospitals, where limited availability of experienced assistants can pose a barrier to efficient robotic surgery [35, 36]. These features in addition to the possibility of telesurgery can support expanding rural MIS access, which was seen as a priority for health system strengthening after the COVID-19 pandemic [37, 38]. These perceived safety-focused and user-friendly design mentioned in the interviews not only benefit the primary surgeon but also could strengthen the overall surgical team dynamic and procedural flow.

The study methodology is limited in its representativeness and data collection tool. Recruited surgeons were sourced from an internal registry and thus only represent a sample of robotic surgeons in the United States. The participants may also hold a favorable view of robotic surgery given that they were early adopters of technology and may have biased the results towards a higher proportion of value-beliefs. Finally, participants who consented and were interviewed made a higher proportion of male, general surgeons and those that practiced in community hospitals; with a notable absence of urologists. This was a limitation of our quasi-random purposing sampling, as opposed to other methods like maximum variation sampling, and consequently these perspectives represent only a subset of views in robotic surgery. In contrast, the timing of the study may have had implications on participants’ views given the early system maturity that limited participants from experiencing all the benefits purported for features that are evolving (e.g. force feedback). The data collection tool used in this study was semi-structured interviews which we did not pilot test and thus may have suffered from subjectivity and researcher bias [39]. This could have impacted questions that probed operational time, efficiency, and clinical outcomes when comparing dV-5 to previous systems or laparoscopic surgery.

This research was a collaborative effort of industry-academic partnership which is both a strength and limitation of the study. All industry personnel who were part of the study were from research rather than commercial/marketing teams, and had expertise as either qualitative researchers or clinician scientists. However, assumptions about the technology may have influenced data collection and analysis. Measures were taken to reduce these limitations including the use of a third-party vendor to recruit participants in an effort to reduce selection bias, application of randomized selection for participants, team consensus around coding structures, and the independent verification of themes during analysis. The senior author who was not an industry member, had final say on data interpretation and manuscript writing. Regardless, the findings of this work would benefit from follow-up studies to validate the themes, especially around clinical benefits, using quantitative studies. Despite the limitations stated, the study provides a cross-sectional and timely snapshot of the different value domains of the new robotic system. This allows for comparisons to be made as the system matures or to triangulate the claims against quantitative evidence. Moreover, the study’s quasi-random sampling allowed for surgeons of various specialties, case volumes and practice types to be interviewed, to the extent possible, which lead to themes being stratified to surface patterns that quantitative data alone would not capture. This also allowed comparison of themes and subthemes across these various domains to contextualize barriers/challenges with adoption across hospital settings and surgeon volumes. Finally, allowing surgeons to relate examples within their practice to themes illustrated how some features may have future benefits that are yet to be realized such as telesurgery, standardized robotic training using benchmarks, and increased access of robotics to rural communities.

Thus, the findings from this study postulates the value of the dV-5 system in surgeon training, improved ergonomics and increasing operational efficiency, while also highlighting the need for further research on clinical outcomes. The qualitative nature of the study adds essential interpretive depth into the practical benefits and barriers surgeons are currently experiencing with the system within their delivery of care.

## Supplementary Information

Below is the link to the electronic supplementary material.

Supplementary Material 1

## Data Availability

De-identified data from this study is available upon reasonable request to the corresponding author.
