# Supplementary material for: Qualitative perspectives of early surgeon users on the value of the daVinci 5 surgical system
Source: J Robot Surg. 2026 May 21;20(1):520. doi: 10.1007/s11701-026-03366-w (PMC13194282; doi:10.1007/s11701-026-03366-w)
Supplement: Supplementary file 1 — Supplementary Material 1 [file 11701_2026_3366_MOESM1_ESM.docx]

**Supplementary materials for manuscript:** Qualitative perspectives of early surgeon users on the value of the daVinci 5 surgical system; Journal of Robotic Surgery; Derek J. Erstad, Zahra A Fazal, Karlis Draulis, Feibi Zheng, Gretchen Jackson, Christy Chai; Associate Professor of Surgery, Baylor College of Medicine, [chirsty.chai@bcm.edu](mailto:chirsty.chai@bcm.edu)

***Supplementary file 1: Interview guide***

Warm-up Questions:

- Could you tell me a little about your role, types of cases you do and experience with robotic surgery, specifically with the daVinci system?

Value pillars of DV-5

- Among the features of the dV-5 system:
  - Which ones have you found valuable and how would you relate it to clinical outcomes.
  - Which ones can you see having a competitive advantage over similar products and why?
- Could you provide examples of any ways you have found the dV5 system positively impacting your practice since implementation, how would you measure or quantify its impact?
- What do you think are future benefits of the dV-5 system that you are not currently using or directly experiencing?
- Any barriers?
- In your view, do the system’s benefits justify its cost? Why or why not?
- If you’ve used Force Feedback, how many cases did you “play” with the force feedback settings before determining your preferences?

Case Insights

Have you used the Case Insights platform before, do you use it regularly? Do you use it on the desktop or just the phone?

1. What have you used Case Insights for:

1. Please tell me more about what features you tried & liked and would need to be improved?
   1. Which features or functions do you use?
   2. Which features or functions are most valuable?
   3. Which features or functions are confusing or not relevant? Why?
   4. What features or functions would you like to see in the future?
   5. Which features or functions should be improved or removed? How or why?

3. What impact/benefits has Case Insights had on your surgical practice or institutional programs?

1. Changes to your surgical technique?
2. Increased efficiency or productivity?
3. Improved clinical outcomes?
4. Enhance teaching or education?
5. Any other benefits?

Is there anything else you would like to tell us about your experience with dV-5 and/or Case Insights?

Closing Statements: Thank you so much for your time and your willingness to share your experience. We will be contacting you soon with information about an honorarium for your time.

***Supplementary file 2: Protocol for analysis and validation***

**APPROACH**

- **Deductive:** Based on a priori codes derived from existing frameworks, theories, or research questions.
- **Inductive:** Open to emergent themes that arise from the data, allowing new insights beyond the initial coding frame.

#### **STAGES**

#### **Phase 1: Familiarization (both authors ZAF and KD)**

- Read and re-read transcripts to immerse yourself in the data in open coding mode
- Take note of patterns, emotions, and contradiction
  - Highlight in red if something needs clarification or is a contradiction [then after discussion we can seek consensus or expert opinion]
  - Highlight in yellow when interviews refer to Case Insights
  - Highlight in other colors [add as necessary]

**Phase 2: Initial Coding (Primary Coder ZAF)**

- Use qualitative analysis software (MAXQDA) and apply:
  - **Deductive codes** from the pre-defined codebook.
  - **Inductive codes** that emerge naturally from the data and make note of these themes back into the codebook with notes.
- Keep a coding log to track decisions and changes to the codebook around any quotes that were in conflict or needed expert consensus (from authors DE/CC) on coding category.

**Phase 3: Validation (Second Coder KD)**

- Provide a random sample (e.g., 10–20%) of transcripts to an independent coder who is blind to the first coder’s work.
- Independent coder uses the same coding protocol:
  - Deductive codes from the shared codebook.
  - Can propose inductive codes – if proposed add to the coding book with parent code link
  - Add comments on sections of quotes where there is a disagreement on coding
  - Correct grammar on quotes using open coding

**Phase 4: Inter-Coder Comparison**

- Compare coded segments:
  - Calculate inter-coder agreement
  - Discuss discrepancies with consensus assessed by an independent reviewer (from authors DE/CC).
  - Revise code definitions or merge/split codes as needed.
- Document changes and rationale in a codebook revision log.

**Phase 5: Theme Development (primary coder ZAF)**

- Identify relationships between codes and themes (parent, child, etc).
- Use data extracts to define and name themes
- Extract relevant quotes for themes
- Begin analysis and develop outputs. Examples include:
  - Co-occurrence heat map
  - Code frequency table

***Supplemental file 3: Heat map showing subtheme frequency stratified by surgeon volume***

|  | **dV5 Volume > Medium** | **dV5 Volume > High** | **dV5 Volume > Low** |
| --- | --- | --- | --- |
| Challenges/barriers > Data infrastructure level > Lag with computing/instruments | 54.55% | 45.45% | 0.00% |
| Challenges/barriers > Data infrastructure level > Unmet Technological Potential | 33.96% | 49.06% | 16.98% |
| Challenges/barriers > Provider level > Not enough clinical evidence | 59.09% | 18.18% | 22.73% |
| Challenges/barriers > Provider level > Interpretability of Case Insights | 41.03% | 30.77% | 28.21% |
| Challenges/barriers > Provider level > Interpretability of FF | 50.00% | 34.62% | 15.38% |
| Challenges/barriers > System level > Anxieties around data | 75.00% | 8.33% | 16.67% |
| Challenges/barriers > System level > learning curve | 43.59% | 33.33% | 23.08% |
| Benefits/value > Surgeon value > Self improvement | 40.00% | 26.67% | 33.33% |
| Benefits/value > Surgeon value > Surgeon autonomy | 33.33% | 38.89% | 27.78% |
| Benefits/value > Surgeon value > Training/mentorship | 38.16% | 34.21% | 27.63% |
| Benefits/value > Surgeon value > Ergonomics | 29.63% | 29.63% | 40.74% |
| Benefits/value > Economic value > OR time | 28.00% | 52.00% | 20.00% |
| Benefits/value > Economic value > Sustainability | 42.11% | 52.63% | 5.26% |
| Benefits/value > Economic value > Complex cases | 50.00% | 25.00% | 25.00% |
| Benefits/value > Economic value > Patient throughput | 28.57% | 71.43% | 0.00% |
| Benefits/value > Economic value > Operational efficiency | 30.43% | 30.43% | 39.13% |
| Benefits/value > Humanistic value > Return to life | 100.00% | 0.00% | 0.00% |
| Benefits/value > Humanistic value > Increased accessibility | 40.00% | 40.00% | 20.00% |
| Benefits/value > Humanistic value > Patient education | 44.44% | 33.33% | 22.22% |
| Benefits/value > Clinical value > Less pain prescriptions | 25.00% | 62.50% | 12.50% |
| Benefits/value > Clinical value > Safety/quality check | 30.77% | 53.85% | 15.38% |
| Benefits/value > Clinical value > Blood loss | 20.00% | 70.00% | 10.00% |
| Benefits/value > Clinical value > LOS | 37.50% | 50.00% | 12.50% |
| Benefits/value > Clinical value > SSI/ tissue tear | 37.04% | 40.74% | 22.22% |
